# Perspectives on the use of aromatherapy from clinicians attending an integrative medicine continuing education event

**DOI:** 10.1186/s12906-019-2572-y

**Published:** 2019-07-12

**Authors:** Amy C. S. Pearson, Susanne M. Cutshall, W. Michael Hooten, Nancy J. Rodgers, Brent A. Bauer, Anjali Bhagra

**Affiliations:** 1 Department of Anesthesia, University of Iowa Carver College of Medicine, Iowa City, IA USA; 2 Department of General Internal Medicine, Mayo College of Medicine, Rochester, MN USA; 3 Department of Anesthesiology & Perioperative Medicine, Mayo College of Medicine, Rochester, MN USA; 4 Department of General Internal Medicine, Mayo College of Medicine, Rochester, MN USA

**Keywords:** Aromatherapy, Essential oils, United States, Physician, Nurse practitioners, Physician assistants, Integrative medicine

## Abstract

**Background:**

The use of essential oils is growing in the United States, but clinician attitudes, experience, and beliefs regarding their use have not previously been studied.

**Methods:**

One hundred five of 106 clinician attendees (99.1%) of an integrative medicine continuing education conference were surveyed using an audience response system to obtain baseline information. Response frequencies of each item were reported. Nonparametric correlations were assessed comparing the statement “In the last 12 months, I have used essential oils for myself and/or my family” with the other agree/disagree statements using Spearman’s rho.

**Results:**

A majority of participants personally used integrative medicine approaches other than aromatherapy (92.6%) and recommended them clinically (96.8%). Most had personally used essential oils (61%) and wished to offer essential oil recommendations or therapies to their patients (74.0%). Only 21.9% felt confident in their ability to counsel patients on safe use. Personal use of essential oils was highly correlated with confidence in the ability to counsel patients on safe use (Spearman coefficient 0.376, *P* = 0.000).

**Conclusions:**

This study indicates that clinicians interested in integrative medicine desire to provide aromatherapy recommendations, but do not feel confident in their ability to do so.

**Electronic supplementary material:**

The online version of this article (10.1186/s12906-019-2572-y) contains supplementary material, which is available to authorized users.

## Background

Aromatherapy is the use of plant-derived essential oils for the purpose of supporting health and wellness. Essential oils are oil-based volatile substances usually produced by water or steam distillation, mechanical expression, or distillation. While essential oils have been used for therapeutic purposes since ancient times, relatively little research has been conducted on their clinical utility in Western medicine. Modern clinicians do not commonly receive formal training on essential oil safety and uses, and counseling on essential oils is not common among medical providers [1]. Although medical and nursing students appear to have positive attitudes toward complementary and alternative medicine (CAM), their sources of information frequently include media sources rather than health professionals, and very little education is provided to them on CAM in their profession core curriculum [2].

Patients often do not disclose their use of integrative or alternative therapies to their clinician, and seek CAM resources outside of the exam room [3–5]. The 2017 Markets and Markets report estimates that the essential oils market value will reach $11.9 billion by 2022, and its compounded annual growth rate from 2017 to 2022 is estimated to be 8.83% [6]. The top 20 essential oils amount to about 104,000 tons of product [7]. The United States consumes about 40% of essential oils, and is also a major importer and exporter of them [7]. The essential oil industry has grown markedly, particularly through direct sales approaches, with major companies reporting a billion dollars in annual sales [8]. There appears to be very little clinically relevant information regarding essential oils published in reputable medical journals. A Google search of the term “essential oils” limited to the United States resulted in about 118,000,000 hits as of April 23, 2019. A PubMed search of “essential oils” and “United States” limited to humans revealed only 61 articles on the same search date.

Essential oil use by the population is widespread, yet little is known about clinicians’ comfort, knowledge base, and attitudes toward essential oil use and patient counseling. While complementary and integrative therapies have been studied in the bedside nursing literature, to our knowledge, there have been no formal surveys published on the attitudes of clinical providers (e.g. physicians, advanced practice nurses, physician assistants) specifically towards aromatherapy [9].

We sought to obtain a baseline understanding of clinicians’ attitudes toward essential oil use for themselves and their patients from those with an interest in Integrative Medicine attending a national conference.

## Methods

The Mayo Clinic Institutional Review Board (IRB) acknowledged that the activity did not require IRB review based on submitted responses to the IRB application (#rev20170511) and was considered exempt. Participation in the survey was voluntary, and no incentive was provided for participation. Attendees to the Mayo Clinic Updates in Integrative Medicine and Health were chosen because they came from a variety of backgrounds (type of degree, primary work facility, and specialty). Clinicians with an interest in integrative medicine were chosen as it was felt that these clinicians would be the most familiar with aromatherapy, and therefore be most likely to offer an informed opinion on its use.

An anonymous electronic survey was utilized to collect responses to questions specific to personal and professional knowledge, attitudes, and use of essential oils by those attending an Integrative Medicine conference.

### Study participants

The participants in this study were clinicians attending a course entitled “Mayo Clinic Updates in Integrative Medicine and Health”. Eligible participants included physicians, physician assistants, nurse practitioners and other non-prescribing providers. One hundred eighteen clinicians registered for the course. One hundred six attendees were present for the survey session, and 105 (99.1%) took the survey. No surveys were excluded due to missing data.

### Study setting

The Continuing Medical Education conference was held at a conference center in November 2017 over the course of 3 days, in which a series of didactic educational lectures and hands-on sessions were delivered throughout the day. Course topics included updates in acupuncture, nutritional supplements, massage therapy, chronic pain, mindfulness, stress & diabetes, integrative cardiology, oncology & women’s health, lifestyle medicine, and humanities in medicine. There were no didactic lectures on aromatherapy, however the participants had the opportunity for face to face discussion with an expert and hands on experience with aromatherapy material after the survey was completed. The conference was planned and delivered by a tertiary referral medical center’s staff as described previously [10, 11].

On the second day of the conference, the attendees were asked to complete a survey of their aromatherapy practices and opinions as well as demographic information using an electronic response system. An audience response system (ARS) was used (Turning Technologies, Youngstown, Ohio) under the direction of a study investigator (ACP) as previously described [12]. Each question was presented on individual slides projected on a main screen. Participants electronically provided their answers on the ARS devices via a lettered keypad. The results were transmitted wirelessly to a central computer and downloaded as a spreadsheet of raw data. Data were linked to individual devices, which were not assigned to specific participants.

### Survey design

No previously validated surveys on aromatherapy were available for reproduction. Survey questions were therefore formulated based on author consensus and previously published studies on clinician attitudes toward complementary and alternative (CAM) or integrative medicine [2, 13–16]. This survey has not previously been published elsewhere and can be found in the Additional file (Additional file 1: “Aromatherapy survey”). The primary goal of the questions was to gain insight on clinicians’ personal knowledge, opinions, and use of essential oils and their perception of patient use of essential oils.

### Data collection

Demographic data collected included age, sex, race, practice location, primary work facility, clinician specialty, clinician type, and previous training in aromatherapy. The primary outcome measure was the participant responses to survey items.

### Statistical analysis

Demographic and survey data were recorded, and the response frequency to each item was analyzed. Nonparametric correlations were assessed comparing the statement “In the last 12 months, I have used essential oils for myself and/or my family” with the other agree/disagree statements using Spearman’s rho. Two-sided tests were used in all analyses, and the level of significance for all statistical tests was set at *P* < 0.05. All data were entered in Microsoft Excel, and analyses were carried out using IBM SPSS Statistics for Windows (Version 21.0, Armonk, NY: IBM Corp. Released 2012). All raw data collected were submitted as Additional file 2.

## Results

### Demographics and practice characteristics

The demographic and practice characteristics contained in Table 1 include age, sex, race, practice location, primary work facility, clinician specialty, clinician type, and previous training in aromatherapy.

**Table 1:** Participant characteristics, *N* = 105

Characteristics	Total Number of Participants, *n* (%)	*N* missing
Age, years		9
25–35	27 (28.1%)	
36–44	38 (39.6%)	
45–54	11 (11.5%)	
55–64	16 (16.7%)	
≥ 65	4 (4.2%)	
Sex		12
Female	78 (83.9%)	
Male	14 (15.0%)	
Other	1 (1.1%)	
Practice Location		10
Urban	26 (27.4%)	
Suburban	18 (19.0%)	
Small City	30 (31.6%)	
Rural	21 (22.1%)	
Primary Work Facility		13
Private Practice	18 (19.6%)	
Academic Center	24 (26.1%)	
Community Health Center	21 (22.8%)	
Other	29 (31.52%)	
Primary Care Provider		11
Yes	64 (68.1%)	
No	30 (31.9%)	
Provider type		11
Advanced Practice Nurse or Physician Assistant	57 (60.6%)	
Physician-in-Training	1 (1.1%)	
Physician	32 (34.0%)	
Registered Nurse	2 (2.13%)	
Other	2 (2.13%)	
Previous Education on Essential Oils or Aromatherapy		9
Yes, programs for clinicians	11 (11.5%)	
Yes, programs for community members	24 (25%)	
No	61 (63.54%)	
Undergone formal training to become an aromatherapist	0 (0%)	14

Eighty-four percent of respondents were female and 35.1% were physicians. The majority practiced in urban (27.4%) or suburban (19.0%) locations. Twenty-six percent worked in an academic center, and 68.1% were primary care providers. Regarding previous training in essential oils, 36.5% reported some training in aromatherapy, and none had undergone training to become a clinical aromatherapist.

### Aromatherapy survey results

A majority of participants agreed with the statements “I consider essential oils to be generally safe when used appropriately” (87.2%), “I believe that there is a need for increased clinician training in the use of essential oils” (75.5%), “I believe that there is a need for more research on the use of essential oils” (89.5%), “I would like to offer essential oil recommendations or therapies to my patients” (74.0%), “In the last 12 months, I have used essential oils for myself and/or my family” (61.0%), “In the last 12 months, I have used integrative medicine approaches other than aromatherapy for myself and/or my family” (92.6%), and “In the last 12 months, I have recommended integrative medicine approaches other than aromatherapy in a clinical setting” (96.8%).

A majority of participants *disagreed* with the statements “In the last 12 months, at least one of my patients has asked me about the use of essential oils for therapeutic purposes” (51.0%), “I feel confident in my ability to counsel patients on the safe use of essential oils for therapeutic purposes” (78.1%) (Table 2).

**Table 2:** Survey responses

Survey Questions	Agree	Disagree	Unsure	N missing		
15. In the last 12 months, at least one of my patients has asked me about the use of essential oils for therapeutic purposes.	47 (49.0%)	49 (51.0%)		9 (8.6%)		
16. I feel confident in my ability to counsel patients on the safe use of essential oils for therapeutic purposes.	21 (21.9%)	75 (78.1%)		9 (8.6%)		
17. I consider essential oils to be generally safe when used appropriately.	82 (87.2%)	1 (1.1%)	11 (11.7%)	11 (10.5%)		
20. I believe that there is a need for increased clinician training in the use of essential oils.	71 (75.5%)	6 (6.4%)	17 (18.1%)	11 (10.5%)		
21. I believe that there is a need for more research on the use of essential oils.	85 (89.5%)	3 (3.1%)	7 (7.4%)	10 (9.5%)		
22. I believe that there is a need for better reimbursement for the clinical use of essential oils.	39 (41.5%)	14 (14.9%)	41 (43.6%)	11 (10.5%)		
23. I would like to offer essential oil recommendations or therapies to my patients.	71 (74.0%)	5 (5.2%)	20 (20.8%)	9 (8.6%)		
24. I would like to undergo training to become certified in clinical aromatherapy.	29 (30.5%)	30 (31.6%)	36 (37.9%)	10 (9.5%)		
25. In the last 12 months, I have used essential oils for myself and/or my family.	58 (61.0%)	37 (39.0%)		10 (9.5%)		
26. In the last 12 months, I have used integrative medicine approaches other than aromatherapy for myself and/or my family.	87 (92.6%)	7 (7.45%)		11 (10.5%)		
27. In the last 12 months, I have recommended integrative medicine approaches other than aromatherapy in a clinical setting.	92 (96.8%)	3 (3.2%)		10 (9.5%)		
	Wellness and relaxation purposes	Treatment of disease	Both wellness & disease treatment	I do not have patients who use essential oils/Never	Unsure	Missing
18. My patients who use essential oils typically use them for	19 (20.4%)	0 (0%)	45 (48.4%)	8 (8.6%)	21 (22.6%)	12 (11.4%)
19. I believe the use of essential oils may be beneficial for	26 (28.0%)	0 (0%)	59 (63.4%)	2 (2.1%)	6 (6.5%)	12 (11.4%)

Regarding patient use of aromatherapy, participants reported that their patients who use essential oils typically used them for wellness and relaxation (20.4%), both wellness and relaxation and treatment of disease (48.4%) or unsure (22.6%). No clinician reported that their patients use essential oils for treatment of disease only.

Regarding their own attitudes toward essential oil use, clinicians believed the use of essential oils may be beneficial for wellness and relaxation (28.0%), both wellness and relaxation and disease treatment (63.4%) or unsure (6.5%). Two percent felt that essential oil use was never beneficial.

### Clinicians’ personal use of essential oils and aromatherapy

Questions were asked specific to personal use of essential oils, and Table 3 contains the correlation of the responses to the statement “In the last 12 months, I have used essential oils for myself and/or my family” with the remaining binary questions of the survey. Clinician use of essential oils was positively and significantly correlated with the following statements: “In the last 12 months, at least one of my patients has asked me about the use of essential oils for therapeutic purposes”, “I feel confident in my ability to counsel patients on the safe use of essential oils for therapeutic purposes”, “I believe that there is a need for increased clinician training in the use of essential oils”, “I believe that there is a need for more research on the use of essential oils”, “I believe that there is a need for better reimbursement for the clinical use of essential oils”, “I would like to offer essential oil recommendations or therapies to my patients”, “I would like to undergo training to become certified in clinical aromatherapy”, and “In the last 12 months, I have used integrative medicine approaches other than aromatherapy for myself and/or my family.” Clinician use of essential oils and aromatherapy was not significantly correlated with the following statements: “I consider essential oils to be generally safe when used appropriately” and “In the last 12 months, I have recommended integrative medicine approaches other than aromatherapy in a clinical setting”.

**Table 3:** Correlation between responses to survey questions and the use of essential oils for self or family in the previous 12 months

Survey Response	Spearman Coefficient	*P*-value
15. In the last 12 months, at least one of my patients has asked me about the use of essential oils for therapeutic purposes.	0.247	0.019*
16. I feel confident in my ability to counsel patients on the safe use of essential oils for therapeutic purposes.	0.376	0.000*
17. I consider essential oils to be generally safe when used appropriately.	0.162	0.155
20. I believe that there is a need for increased clinician training in the use of essential oils.	0.387	0.001*
21. I believe that there is a need for more research on the use of essential oils.	0.252	0.021*
22. I believe that there is a need for better reimbursement for the clinical use of essential oils.	0.639	0.000*
23. I would like to offer essential oil recommendations or therapies to my patients.	0.387	0.001*
24. I would like to undergo training to become certified in clinical aromatherapy.	0.522	0.000*
26. In the last 12 months, I have used integrative medicine approaches other than aromatherapy for myself and/or my family.	0.294	0.005*
27. In the last 12 months, I have recommended integrative medicine approaches other than aromatherapy in a clinical setting.	−0.143	0.178

*P < 0.05

## Discussion

In the United States, it is estimated that 62% of adults have used some form of complementary or alternative therapy in the last 12 months [17], although adult use specifically of essential oils or aromatherapy is unknown. A recent Swiss study estimated that 10.7% of patients with chronic low back pain used aromatherapy and rated it a 4.2 out of a 10-point scale for usefulness [18]. Other studies have shown that aromatherapy may have health benefits, including relief from anxiety and depression, improved quality of life, improved sleep, and pain relief for knee osteoarthritis and renal colic [19–25].

In this study of US clinicians interested in integrative medicine, nearly all clinicians used integrative therapies for themselves and their family (92.6%) and recommended them to patients (96.8%). However, only 61% personally used aromatherapy for themselves or their families, and only 11.5% had any kind of clinician oriented education on essential oils. While most clinicians felt that essential oil use was generally safe and wished to offer essential oil recommendations or therapies to patients, only 21.9% felt confident in their ability to counsel patients on their safe use. A similar gap was found in a previous study, where only 15.1% of nursing students and 7.1% of medical students reported having enough knowledge about aromatherapy, while 96.3% of nursing students and 88.9% of medical students considered it useful or were undecided [2].

Personal use of essential oils was positively correlated with the belief that there is a need for increased training, research, and reimbursement regarding their use, and a desire to offer essential oil recommendations and receive formal certification in this modality. Personal use was also correlated with confidence in counseling patients as well as being approached by patients regarding aromatherapy.

This study indicates that clinicians interested in integrative medicine are being approached by patients about aromatherapy use. Clinicians desire to provide recommendations, but do not feel confident in their ability to do so. As the essential oil market continues to grow within the US and the world, clinician expertise in essential oil use may be increasingly valued by both patients and healthcare systems, and could possibly lead to safer and more evidence-based use. Healthcare systems have already begun aromatherapy programs, which include clinician education regarding the evidence for aromatherapy and essential oils, selection and properties of oils, and their safe use [26, 27]. We feel that increasing clinicians’ familiarity and knowledge base, with adequate structure and support from employers and hospitals, may help to bridge this gap. At the Mayo Clinic, the authors (SC & NR) and their colleagues developed an aromatherapy program for pediatric patients. In this model, key stakeholders were included early, and staff education and support services were implemented prior to the program’s start. Essential oils were chosen based on their safety profile and were only used for inhalation. As a result, 76.7% of nurses administered aromatherapy to their patients at least monthly, and 89% rated the benefit from aromatherapy as 4 or above on a scale of 0–10 (ten being greatest benefit) [26]. In the future, programs such as this could expand to the outpatient realm, where clinicians are given tools to offer advice on safe use of basic essential oils for certain clinical situations.

This is the first US study of clinician attitudes, practices, and beliefs regarding essential oils and aromatherapy. However, this study is limited in that it was a small study of clinicians of heterogeneous educational backgrounds, practice types, and locations. All clinicians were attending a multi-day course in Integrative Medicine, and were therefore more likely to have familiarity with and favorable opinions of integrative treatments such as aromatherapy. While most questions pertained to participants’ opinions, questions asking objective data may have been subject to recall bias. We attempted to mitigate this by limiting recall to a finite time period (12 months). It is also possible that clinicians are not asking about or aware of their patients’ use, and extent of use, of essential oils. For these reasons, we offered the “unsure” answer option for a more accurate representation.

The effect of essential oil use on the health and safety of the American public is not known, and the influence of knowledgeable clinicians on safe and appropriate use is also unknown. Future research is needed to determine the nationwide prevalence of essential oil use by patients and clinicians, and the frequency and accuracy of clinician counseling regarding aromatherapy use.

## Conclusions

This study suggests that clinicians interested in integrative medicine wish to increase their aromatherapy knowledge in order to provide evidence-based guidance to their patients, but do not feel confident in their ability to do so.

## Additional Files

Additional file 1: *Aromatherapy Survey *The survey as presented to the participants. (PDF 282 kb)

Additional file 2: *Aromatherapy Perspectives – original dataset *This is the raw dataset of the survey answers. (XLSX 34 kb)

## Abbreviations

ARS: Audience Response System
CAM: Complementary & Alternative Medicine
IRB: Institutional Review Board
US: United States

## References

[1] Maddocks-Jennings W, Wilkinson JM (2004). Aromatherapy practice in nursing: literature review. J Adv Nurs.

[2] Yildirim Y, Parlar S, Eyigor S, Sertoz OO, Eyigor C, Fadiloglu C (2010). An analysis of nursing and medical students' attitudes towards and knowledge of complementary and alternative medicine (CAM). J Clin Nurs.

[3] Galicia-Connolly E, Adams D, Bateman J, Dagenais S, Clifford T, Baydala L (2014). CAM use in pediatric neurology: an exploration of concurrent use with conventional medicine. PLoS One.

[4] Misra SM, Guffey D, Roth I, Giardino AP (2017). Complementary and alternative medicine use in uninsured children in Texas. Clin Pediatr (Phila).

[5] Shakeel M, Little SA, Bruce J, Ah-See KW (2007). Use of complementary and alternative medicine in pediatric otolaryngology patients attending a tertiary hospital in the UK. Int J Pediatr Otorhinolaryngol.

[6] marketsandmarkets.com. Essential Oils Market by Product Type (Orange, Lemon, Lime, Peppermint, Citronella, Jasmine), Method of Extraction, Application (Food & Beverage, Cosmetics & Toiletries, Aromatherapy, Home Care, Health Care), and Region - Global Forecast to 2022 2017 [updated June 2017. Report code FB 5385:[Available from: https://www.marketsandmarkets.com/Market-Reports/essential-oil-market-119674487.html.

[7] Devi MP, Chakrabarty S, Ghosh SK, Bhowmick N, Sharangi AB, Datta S (2015). Essential oil: its economic aspect, extraction, importance, uses, hazards and quality. Value addition of horticultural crops: recent trends and future directions.

[8] Monroe R. How essential oils became the cure for our age of anxiety. The new Yorker [Internet]. 2017 October 31, 2018; (October 9, 2017). Available from: https://www.newyorker.com/magazine/2017/10/09/how-essential-oils-became-the-cure-for-our-age-of-anxiety

[9] Boesl R, Saarinen H (2016). Essential oil education for health care providers. Integr Med (Encinitas).

[10] Hooten WM, Bruce BK (2011). Beliefs and attitudes about prescribing opioids among healthcare providers seeking continuing medical education. J Opioid Manag.

[11] Pearson AC, Eldrige JS, Moeschler SM, Hooten WM (2016). Opioids for chronic pain: a knowledge assessment of nonpain specialty providers. J Pain Res.

[12] Pearson AC, Moman RN, Moeschler SM, Eldrige JS, Hooten WM (2017). Provider confidence in opioid prescribing and chronic pain management: results of the opioid therapy provider survey. J Pain Res.

[13] Abbott RB, Hui KK, Hays RD, Mandel J, Goldstein M, Winegarden B (2011). Medical student attitudes toward complementary, alternative and integrative medicine. Evid Based Complement Alternat Med.

[14] Kanadiya MK, Klein G, Shubrook JH (2012). Use of and attitudes toward complementary and alternative medicine among osteopathic medical students. J Am Osteopath Assoc.

[15] Lie D, Boker J. Development and validation of the CAM health belief questionnaire (CHBQ) and CAM use and attitudes amongst medical students. BMC Med Educ. 2004;4(2). 10.1186/1472-6920-4-2 PMC373452 14718061

[16] Freymann H, Rennie T, Bates I, Nebel S, Heinrich M (2006). Knowledge and use of complementary and alternative medicine among British undergraduate pharmacy students. Pharm World Sci.

[17] Barnes PM, Powell-Griner E, McFann K, Nahin RL. Complementary and alternative medicine use among adults: United States, 2002. Adv Data. 2004;(343):1–19. 15188733

[18] Dubois J, Scala E, Faouzi M, Decosterd I, Burnand B, Rodondi PY (2017). Chronic low back pain patients' use of, level of knowledge of and perceived benefits of complementary medicine: a cross-sectional study at an academic pain center. BMC Complement Altern Med.

[19] Sanchez-Vidana DI, Ngai SP, He W, Chow JK, Lau BW, Tsang HW. The effectiveness of aromatherapy for depressive symptoms: a systematic review. Evid Based Complement Alternat Med 2017;2017:5869315. 10.1155/2017/5869315 PMC5241490 28133489

[20] Press-Sandler O, Freud T, Volkov I, Peleg R, Press Y (2016). Aromatherapy for the treatment of patients with behavioral and psychological symptoms of dementia: a descriptive analysis of RCTs. J Altern Complement Med.

[21] Nasiri A, Mahmodi MA, Nobakht Z (2016). Effect of aromatherapy massage with lavender essential oil on pain in patients with osteoarthritis of the knee: a randomized controlled clinical trial. Complement Ther Clin Pract.

[22] Lakhan SE, Sheafer H, Tepper D. The effectiveness of aromatherapy in reducing pain: a systematic review and meta-analysis. Pain Res Treat 2016;2016:8158693. 10.1155/2016/8158693 PMC5192342 28070420

[23] Irmak Sapmaz H, Uysal M, Tas U, Esen M, Barut M, Somuk BT (2015). The effect of lavender oil in patients with renal colic: a prospective controlled study using objective and subjective outcome measurements. J Altern Complement Med.

[24] Hwang E, Shin S (2015). The effects of aromatherapy on sleep improvement: a systematic literature review and meta-analysis. J Altern Complement Med.

[25] Bystritsky A, Hovav S, Sherbourne C, Stein MB, Rose RD, Campbell-Sills L (2012). Use of complementary and alternative medicine in a large sample of anxiety patients. Psychosomatics..

[26] Conlon PM, Haack KM, Rodgers NJ, Dion LJ, Cambern KL, Rohlik GM (2017). Introducing essential oils into pediatric and other practices at an Academic Medical Center. J Holist Nurs.

[27] Joswiak D, Kinney ME, Johnson JR, Kolste AK, Griffin KH, Rivard RL (2016). Development of a health system-based nurse-delivered aromatherapy program. J Nurs Adm.

