# Supplementary material for: Perspectives on the use of aromatherapy from clinicians attending an integrative medicine continuing education event
Source: BMC Complement Altern Med. 2019 Jul 12;19:174. doi: 10.1186/s12906-019-2572-y (PMC6625079; doi:10.1186/s12906-019-2572-y)
Supplement: Supplementary file 1 — *Aromatherapy Survey *The survey as presented to the participants. (PDF 282 kb) [file 12906_2019_2572_MOESM1_ESM.pdf]

Clinician perspectives on the use of essential oils and aromatherapy in the United States

A. Pearson, S. Cutshall, N. Rodgers, B. Bauer, A. Bhagra

Mayo Clinic Integrative Medicine Conference
Wisconsin Dells, Wisconsin
November 9-11, 2017

Please participate in a short, introductory survey

- Who: All attendees
- What: A brief survey via the audience response system
- When: The next 10 minutes
- Why: To learn about your perspectives on the use of clinical aromatherapy

- Responses are anonymous
- Your participation is voluntary but appreciated
- There are no known risks to participating

MAYO CLINIC

Definitions

- **Aromatherapy**: the use of essential oils for therapeutic purposes
- **Essential oil**: a volatile oil typically obtained by distillation and having the characteristic fragrance of the plant from which it is extracted
- **Clinical**: relating to the treatment of patients
- **Aromatherapist**: typically a professional who has become certified in the use of essential oils through a NAHA-approved course.

What is your age?

A) 25-35
B) 36-44
C) 45-54
D) 55-64
E) >65

With what gender do you identify?

A) Male
B) Female
C) Other

What is your race?

A) African American/black
B) Hispanic/Latino
C) American Indian or Alaska Native
D) Asian
E) Native Hawaiian or Pacific Islander
F) Caucasian/white
G) Biracial/multiracial
H) Other

What is your practice location?

A) Urban
B) Suburban
C) Small city
D) Rural

In what type of facility do you primarily work?

A) Independent practice
B) Academic center
C) Community health center
D) Government facility
E) Other

Are you a primary care provider (internal medicine, family medicine, pediatrics, general OB/GYN)?

A) Yes

B) No

MAYO CLINIC

What type of clinician are you?

A) Advanced practice nurse or Physician assistant
B) Physician-in-training (resident, fellow, medical student)
C) Physician
D) Registered nurse
E) Complementary medicine practitioner: Acupuncturist, Massage therapist, Chiropractor, etc.
F) Other (hospital administrator, attorney)

Base your answer on your primary specialty

Apart from this event, have you received education on the use of essential oils or aromatherapy?

A) Yes, through programs designed for clinicians
B) Yes, through programs designed for community members
C) No

Have you undergone a formal training course to become a certified clinical aromatherapist?

A. Yes
B. No

Please answer the following questions based on your personal experience and opinions

MAYO CLINIC

In the last 12 months, at least one of my patients has asked me about the use of essential oils for therapeutic purposes.

A. Agree
B. Disagree

I feel confident in my ability to counsel patients on the safe use of essential oils for therapeutic purposes.

A. Agree
B. Disagree

MAYO CLINIC

I consider essential oils to be generally safe when used appropriately.

A. Agree
B. Disagree
C. Unsure

MAYO CLINIC

My patients who use essential oils typically use them…

A. For wellness and relaxation purposes
B. For the treatment of disease
C. Both A and B
D. I do not have patients that use essential oils
E. Unsure

I believe the use of essential oils may be beneficial…

A. For wellness and relaxation purposes
B. For the treatment of disease
C. Both A and B
D. Never
E. Unsure

I believe that there is a need for increased clinician training in the use of essential oils.

A. Agree
B. Disagree
C. Unsure

MAYO CLINIC

I believe that there is a need for more research on the use of essential oils.

A. Agree
B. Disagree
C. Unsure

MAYO CLINIC

I believe that there is a need for better reimbursement for the clinical use of essential oils.

A. Agree
B. Disagree
C. Unsure

MAYO CLINIC

I would like to offer essential oil recommendations or therapies to my patients.

A. Agree
B. Disagree
C. Unsure

MAYO CLINIC

I would like to undergo training to become certified in clinical aromatherapy.

A. Agree
B. Disagree
C. Already certified
D. Unsure

In the last 12 months, I have used essential oils for myself and/or my family.

A. Agree
B. Disagree

In the last 12 months, I have used integrative medicine approaches *other than aromatherapy* for myself and/or my family (acupuncture, massage, chiropractic, etc.).

A. Agree
B. Disagree

In the last 12 months, I have recommended integrative medicine approaches *other than aromatherapy* in a clinical setting (acupuncture, massage, chiropractic, etc.).

A. Agree
B. Disagree

MAYO CLINIC

MAYO
CLINIC

Thank you for your attention!

Amy-Pearson@uiowa.edu with any questions
